# Account of an Epidemic of Small-Pox in Jamaica in 1851-2

**Published:** 1858-04

**Authors:** Edward Cator Seaton

**Affiliations:** Hon. Secretary to the Small-Pox and Vaccination Committee.


### TRANSACTIONS

OF THE

### EPIDEMIOLOGICAL SOCIETY OF LONDON.

---

### Papers and Communications.

---

### ACCOUNT OF AN EPIDEMIC OF SMALL-POX IN JAMAICA IN 1851-2.

By EDWARD CATOR SEATON, M.D., Hon. Secretary to the Small-Pox and Vaccination Committee.

[*Read before the Society, July 2nd*, 1855.]

On the 14th of April, 1851, an emigrant ship, the *Brandon*, sailed from Sierra Leone with a crew of 32 men, and having on board 249 African emigrant free labourers. Some of these Africans had recently been taken from a Congo slaver, in which, as is very common, a good deal of small-pox had existed; but there was no appearance of sickness among any of them when they were received on board the *Brandon*. On the 17th, three days after leaving Sierra Leone, one of the Africans, a boy about 14 years of age, was attacked with small-pox, another on the 23rd, and a third on the 27th. The first and third cases were in unprotected individuals, and both of them confluent and fatal. In the second, the patient, a boy, who had been vaccinated, had a mild attack, and recovered in nine or ten days. They were isolated, as far as was possible on a crowded ship, and no other case occurred.

The ship arrived at Port Royal, Jamaica, on the 13th of May, six days after the last death from small-pox. She remained in quarantine at Port Royal till the 31st, when she proceeded to Falmouth, on the north side of the island, where she landed the Africans. These were then distributed over different properties in Trelawney, and continued healthy until the beginning of July, when two of them, unvaccinated boys residing on the Hyde Estate, were attacked with small-pox simultaneously; from them other residents on the estate became affected, and the disease subsequently spread over the whole island.

It is worthy of observation that these lads had been two months in the island, apparently quite well, before they manifested signs of the disease; and that small-pox, though not previously in Jamaica, was existing, and had been existing, for many months, in several of the other West Indian islands, as Barbadoes, St. Lucia, Montserrat, St. Kitt's, and Granada. It seems difficult to suppose that in these boys there could have been so protracted a period of incubation, and their simultaneous attack leads naturally to the suspicion that the variolous poison might have been retained by some articles of clothing, etc., on the exposure of which to air it had been given forth, and thus developed the disease. But Dr. Gavin Milroy, to whose excellent report on the general sanitary condition of Jamaica I am indebted for the foregoing facts, expressly states, that in the case of these African lads there could have been no fomites, and that they had no raiment whatever, except the partial clothing on them.

However this may be, it was in them and on the Hyde Estate that the epidemic of small-pox in Jamaica undoubtedly had its origin, and from this source it may be traced over the island. In September, 1851, it broke out in Duncan's district, Trelawney, and was distinctly traced to communication with the Hyde Estate. In November, 1851, we find it in St. Ann's, adjoining Trelawney to the east; and in December 1851, at Montego Bay in St. James's, joining Trelawney to the west. It existed also by the end of 1851 in Portland, at the eastern part of the island; how conveyed, I have no information. In the spring of next year it is found at Kingston and Spanish Town; the first cases in Spanish Town being distinctly traceable to communication with Falmouth in Trelawney, where the disease still remained. A little later we find it at Metcalf, where it originated from three different sources of contagion, the first case having occurred at Kendal, from communication with St. Ann's; the second at Annoto Bay, from communication with Portland; and the third, at the same place, from communication with Kingston. About the same time it began in Clarendon and Vere; and the last account we have of it in the papers intrusted to me, was in September 1852, when it was just beginning to appear at Savanna-la-Mar in Westmoreland, having been imported there from Lucia in Hanover, where it had already been some time prevalent.

In the spring of 1852, the Central Board of Health of Jamaica issued to the medical practitioners practising in the island, a paper of questions, seventy-nine in number, intended to elicit from them the facts they had observed, and the opinions they had formed with regard to the extent to which vaccination had been practised in the island; the mode in which it was performed; the success which attended the operation, and its protective value against small-pox in different races and various classes of society; the sources of the lymph employed; the usual mode of transmitting it, and other points connected with vaccination generally; with regard to the origin, character, progress, and effects of the present epidemic in the various towns and districts which it invaded; and with regard to the practice of inoculation. Sixteen medical practitioners returned answers to the questions thus submitted to them, and several others forwarded to the Central Board general information on the subjects of inquiry; and Dr. Milroy, believing that this information would form a useful contribution to the general investigation into the subject of small-pox and vaccination, undertaken by the Epidemiological Society, was kind enough to procure and forward to me copies of all these returns. They carry the history of the epidemic only up to the latter part of the year 1852, at which time it was still existing in the island, and, indeed, as I have already observed, was only commencing its invasion in some portions of it. I am not aware whether any subsequent returns were made to the Central Board. Although I am not able, therefore, to lay before the society a complete account of the epidemic, I find in these papers so much important and interesting matter, that I have thought it right to undertake an analysis of them, and to bring the results before the notice of the society. On the present occasion, however, I shall confine myself to the history of the epidemic of 1851-2, and to the facts illustrative of the protective value of vaccination, reserving the information respecting the operation of vaccination itself, the progress of the vaccine disease in hot climates, and various questions connected therewith, for another communication, which I shall hope to have the honour of presenting at some future time.

When the small-pox broke out in Jamaica in July 1851, b 2

it found everything in the island favourable for its reception and propagation. There had been no epidemic of the disease for twenty years, and thus there was almost an entire generation ready to receive it, unless, indeed, they had taken the precaution to protect themselves by vaccination. This there is abundant proof that they had not done; and though there are no data which enable me to state statistically the proportion of vaccinated and unvaccinated, there can be no difficulty in affirming that the great majority of the population were, at the time the disease first broke out, among the latter. A proof of this is afforded by the numbers brought to the various medical men to be vaccinated on the outbreak of the epidemic. Thus Dr. Bowerbank, of Spanish Town, states that he vaccinated, in the early portion of 1852, about 2,450. Dr. Brooks who, in 1847, made vain attempts to induce the people in the parish of Manchester, in which he then resided, to be vaccinated, in five weeks of 1851 operated on upwards of 1,500 people. Dr. Thomas Clarke of Annoto Bay had vaccinated, during sixteen years' residence, about 200 persons, but since the outbreak of the epidemic above 3,000. Dr. Jelly of Westmoreland, in a residence of twenty-two years, had vaccinated *none* till within six weeks of his report, in which time he had operated on 2,000. Dr. Adolphus, in five years residence at Savanna-la-Mar, had had two formal vaccinations, at which, by great persuasion, he could only get about 200 people to attend, but latterly he has vaccinated above 1,500. The testimony of other medical men is to the same effect, and proves that, in Jamaica, as here, in the absence of some constraining law, or of the fear of an immediate attack of the disease, the people habitually neglect the protection to which they readily resort when danger is at hand, but often then not until it is too late.

In a population so circumstanced, it is not to be wondered at that the disease should have been widely diffused and very fatal; and, as if the natural sources of diffusion were not sufficient, we find that in some parts of the island it was propagated by inoculation, till by an Order in Council the practice was made penal. There are no means of determining the exact ratio of attacks to population, nor the total number of deaths,—and very many, indeed, of these occurred where there was no medical assistance whatever.

The epidemic does not appear to have manifested any special peculiarities of character. The period of incubation is stated generally as about twelve days. Dr. Bowerbank says, the shortest time he noticed was twelve days, the longest twenty-two or twenty-three, and he places the average at sixteen. The eruption, according to the testimony of all the observers, generally appeared on the third day of fever. "In several," says Dr. Bowerbank,—to whose report I shall have again and again to refer, and of which I cannot speak too highly for its elaborate care and precision—"on the fourth, in a few on the second, in a few on the fifth. In one remarkable case the eruption did not come out till twenty-two days after the first appearance of fever, and in this case the period of incubation was also protracted. It occurred in a young coloured child, whose brother, in the same house, had the disease naturally." Dr. Clarke, of Annoto Bay, remarks, as the result of his own observation, that there was frequent deviation from the period ordinarily assigned for the duration of the precursory or eruptive fever, and this he attributes to the malaria prevalent in the district in which he practises.

The immense majority of cases observed were in the negro race; but not only does this race form an overwhelming portion of the population, but it is by them especially that vaccination has been neglected. Dr. Clarke, who estimates the cases which occurred in his district at about 500, says, that there was not one white person among them, *because the whites were universally vaccinated.* For the same reason, also, there were very few cases in the coloured race. Mr. Kingdon of St. Ann's, who speaks of between four and five hundred cases having occurred in his district, in like manner mentions, that not more than half a dozen of them were white persons, and for a similar reason. None of the reporters countenance the notion that there is more disposition in the negro race to take the disease than there is in white people who are unprotected; nor, *so far as observation of the present epidemic goes,* that there is any particular tendency in the negro to the malignant form of the disease. Indeed, Dr. Bowerbank mentions that the worst cases seen by him were in whites. Where malignant small-pox was observed in the persons of negroes, it was attributed by most reporters to extraneous causes, as their filth, misery, and the like, and not to anything inherent in their constitution.

In cases which were fatal, death generally took place during the secondary fever, from the tenth to the nineteenth day from the commencement of the eruption, unless the case were malignant, when it generally occurred earlier. Dr. Bowerbank especially noted the period at which death took place in an infant and thirteen adults who died out of 101

cases treated in the small-pox hospital. The infant died on the sixth day; one adult on the tenth, one on the twelfth, one on the fourteenth, two on the fifteenth, one on the sixteenth, two on the seventeenth, one on the eighteenth, one on the nineteenth, one on the twenty-second, one on the twenty-third, and one on the thirty-second day.

"The variola," according to Dr. Bowerbank, "was accompanied in several cases with profuse salivation and severe affection of the fauces; the worst case of this kind being in a person who had previously been vaccinated, the only one of a large family (all vaccinated) who took the disease. The most frequent complication was dysentery; in those who had suffered lately from measles it assumed the form of diarrhœa. Pneumonia occurred in a few, and proved fatal to one or two young children. Menorrhagia occurred in a few, also hæmaturia. Boils were frequent during convalescence, but these were epidemic during the whole year. Ophthalmia and ulceration of the cornea took place in many. In three cases there was phrenitis; in one, general paralysis; in several, convulsions; in several, nervous irritation from destruction of the skin." Dr. Brooks and Dr. Clarke concur, as well as other observers, in mentioning diarrhœa and dysentery as the most frequent complications; but in most cases, Dr. Clarke observes, the condition of the skin alone accounted for the severity and danger of the secondary fever. Dr. Chamberlaine, of Kingston, mentions as sequelæ, severe ophthalmia, boils, abscesses, and destruction of the cutis vera. Dr. Lawson, of St. James's, especially mentions the numbers who, after the epidemic was over, came to the town in which he lives (Montego Bay) for advice, in whom one or both eyes had been destroyed.

Dr. Bowerbank saw one case in which small-pox occurred during the progress of measles; the case was very severe, and the young woman (coloured) died on the fourth or fifth day of the measley eruption. Small-pox succeeded to measles in several cases, and in such the disease was always severe. Dr. Chamberlaine of Kingston saw the same concurrence of small-pox with measles.

Dr. Bowerbank and Dr. Brooks, of Spanish Town, and Dr. Chamberlaine of Kingston, concur in stating that they saw no cases of varicella during the epidemic; but Dr. Bowerbank says, that several of the cases of modified small-pox seen by him were the *variola varicelloides* of Rayer, which, in another part of his communication, he describes as marked by conoidal *unumbilicated* pustules. All these cases had been vaccinated. Nor do I find any statement throughout these returns, of varicella having been prevalent during the period of the small-pox epidemic. My friend Dr. Milroy, however, in the report to which I have alluded, speaks of it as having existed in various parts of the island; and I cannot but think that many cases of it must have been confounded with cases of small-pox, when I find several of the practitioners stating, that the mortality they observed from the latter disease was not above six per cent.

There is nothing particular observed on the subject of treatment. The necessity of cleanliness and free ablution is strongly insisted on; the use of anodynes to allay irritation, and of stimulants in the stage of maturation, is highly commended, and of all stimulants beer is the most prized. Dr. Clarke observes on this point, "Brandy and wine completely failed in my hands in secondary fever of the typhoid type; but the effect of bottled pale ale surpassed any expectations I could have formed, and I do not exaggerate when I state that I came, after the trial of it, to the treatment of the condition just mentioned (typhoid fever) with as much satisfaction and confidence, as I had previously experienced of dissatisfaction and all but despair." It may be interesting to add the popular mode of treatment, which was, to strip the patient naked, roll him up in plantain leaves, cover him over with a piece of thin muslin, and give him a decoction of corn-stalks to drink; the pustules were opened early, and the bodies were daily smeared with ashes and water.

I now approach the important subject of the influence exercised over the epidemic by vaccination. That a considerable number of cases of small-pox occurred in persons who had undoubtedly been successfully vaccinated, the facts I shall have hereafter to adduce will show. But a large number, also, took place in persons *reputed* to have been vaccinated, but who had never really gone through the vaccine disease. Great as is the difficulty of getting the negro population to submit to the operation at all, except under the pressure of terror, it is still greater to induce them to bring their children for inspection, that the success of the vaccination may be determined. Dr. Brooks says on this point, that of fifty or sixty people vaccinated, sometimes not one will return. Dr. McGrath complains that numbers do not return. Dr. Lawson of Montego Bay, who had vaccinated 1340 persons since the outbreak of the epidemic, states, that he was only able to ascertain the result of the operation in a very few cases, comparatively speaking. It is obvious, therefore, that a large number of persons pass as vaccinated who have not been really protected. Dr Clarke, of Annoto Bay, gives some excellent illustrations. "Of 265 persons," he says, "named in my list as subjected to vaccination in this town, where small-pox has been prevalent for the last four or five months, one only, Elizabeth Francis, has taken the disease, and she is happily a living witness to declare that no vesicle followed the operation in her case. It would be tedious to detail the several instances in which vaccination has been stated, and with an extraordinary amount of circumstantiality, to have failed as a protection against small-pox. Suffice it to say, that each and all have, in succession, broken down, every case having proved, on investigation, to be either one of some other eruptive disorder, or one in which the vaccine vesicle had never developed itself, or one in which the vaccine puncture had never been made." Another example is afforded on the Gibraltar Estate in the same parish, where, out of 113 persons vaccinated, four were stated to have subsequently had small pox. "In three out of the four," says Dr. Clarke, "I possess unequivocal proof that the vaccine vesicle never developed itself, and the fourth I have not yet been able to find out." Again, of eighty-nine persons vaccinated on the estate of Iter Boreale, three were stated to have had small-pox subsequently. In two there was clear proof that no vaccine vesicle had ever developed itself; the third person could not be found. "Results," he adds, "might be given exemplifying the all but complete immunity of whole localities where the vaccine operation has been more fully carried out, *e. g.* Belfield Pen, Agualta Vale Estate, etc.; but the protection against small-pox which vaccination furnishes, is best illustrated in districts such as those first named, where its use has been more partial, and where, in consequence, the subjects of vaccine and the victims of small-pox have been in habits of intercourse more or less habitual; and here the result is, that, setting aside six cases of vaccine puncture, in which it is already ascertained that no vesicle followed, and two others into which circumstances for the present prevent inquiry, we have out of 459 persons subjected to the vaccine puncture, and residing in localities which have been the seat of epidemic small-pox for months past, not one instance of that disease."

If to those cases in which vaccination was followed by the development of no vesicle at all, we add the cases in which it produced a spurious result, a considerable further deduction will have to be made from the total number of cases stated to be after vaccination. There is abundant evidence in the returns that vaccination was often performed without due regard to those precautions which are essential to its success. Dr. Lawson says, that he feels no surprise that small-pox should have occurred after what in many instances was called vaccination, when in some of them he observed suppuration going on on the third or fourth day after the operation; and in others, he has been told, at the expiration of a little more than that space of time, the fluid was "ripe and yellow," and was considered fit, and actually used, for vaccination; and Dr. Donald, of St. Ann's and St. Mary's, says, that impure lymph was largely disseminated through the parish, for several months, by quacks employed by the local Board, causing in many instances violent local disturbance without, of course, any protective result.

It is important evidence of the power of effectual vaccination to prevent small-pox, that not one of the medical practitioners in the island saw a single case of that disease in a person vaccinated by himself. Dr. Lawson expressly states, that he saw no case in which there was any authentic proof of vaccination at all. Of a hundred and twenty-five patients who were admitted into the small-pox hospital at Kingston, in the thirteen weeks following the 6th of June, 1852, there was not one who had been vaccinated. Dr. Raphey, in a report to the Local Board of St. George's, mentions his having vaccinated thirty-seven children at the parochial school at Buff Bay. The school being subsequently closed, the children were sent home to their relations in the neighbourhood, where the disease was very malignant and fatal; but all these thirty-seven children are living, and were protected by their vaccination.

The most important evidence with regard to the *modifying* power of vaccination is given by Dr. Bowerbank of Spanish Town, who has furnished the register of 301 cases of small-pox which fell under his observation. From this register I have constructed the Table given on the next page. Dr. Bowerbank's report is particularly valuable, because he has distinguished the various races.

It will be seen that of the 301 patients, 241 had never been vaccinated, and that of these 45 died, or upwards of eighteen per cent.; while of 58 who had been vaccinated, only two died, and of two who had been inoculated, one died. Of the two deaths after reputed vaccination, one was in a woman who showed no cicatrix whatever; the other was in a young man who had previously had measles, and was attacked with dysentery, which was in reality the disease of which he died.

TABLE SHOWING THE MODIFYING INFLUENCE OF VACCINATION.
(*From Dr. Bowerbank's Report.*)

| | Cases. | Deaths. | Discrete. | | Confluent. | | Modified. | |
|---|---|---|---|---|---|---|---|---|
| | | | Cases | Dths. | Cases | Dths. | Cases | Dths. |
| UNVACCINATED. | | | | | | | | |
| In hospital: White . . . | 0 | 0 | | | | | | |
| Coloured . | 13 | 1 | 67 | 2 | 13 | 10 | 1 | 0 |
| Black . . . | 68 | 11 | | | | | | |
| Parochial: White . . . | 0 | 0 | | | | | | |
| Coloured . | 35 | 6 | 60 | 6 | 13 | 5 | 3 | 0 |
| Black . . . | 41 | 5 | | | | | | |
| Private: White . . . | 5 | 1 | | | | | | |
| Coloured . | 50 | 11 | 56 | 5 | 25 | 16 | 3 | 1 |
| Black . . . | 29 | 10 | | | | | | |
| Total . . . . . . . | 241 | 45 | 183 | 13 | 51 | 31 | 7 | 1 |
| VACCINATED. | | | | | | | | |
| In hospital: White . . . | 0 | 0 | | | | | | |
| Coloured . | 5 | 0 | 12 | 1 | 2 | 1 | 6 | 0 |
| Black . . . | 15 | 2 | | | | | | |
| Parochial: White . . . | 1 | 0 | | | | | | |
| Coloured . | 4 | 0 | 9 | 0 | 1 | 0 | 2 | 0 |
| Black . . . | 7 | 0 | | | | | | |
| Private: White . . . | 4 | 0 | | | | | | |
| Coloured . | 15 | 0 | 13 | 0 | 2 | 0 | 11 | 0 |
| Black . . . | 7 | 0 | | | | | | |
| Total . . . . . . . | 58 | 2 | 34 | 1 | 5 | 1 | 19 | 0 |
| INOCULATED. | | | | | | | | |
| White . . . . . . . . . . . . . | 1 | 1 | 0 | 0 | 2 | 1 | 0 | 0 |
| Coloured . . . . . . . . . . . | 1 | 0 | | | | | | |
| Total . . . . . . . | 2 | 1 | 0 | 0 | 2 | 1 | 0 | 0 |

| Period of Life. | Vaccinated. | | Unvaccinated. |
|---|---|---|---|
| Adults - - - - | - 42 | .... | 140 |
| Children, *i.e.* from 3 to 14 years old | - 15 | .... | 69 |
| Infants - - - - | - 1 | .... | 32 |
| | 58 | .... | 241 |

Of the vaccinated, there were 34 who exhibited characteristic marks; 12 who had but imperfect marks; and 12 in whom there were no cicatrices at all. Of the 241 who had not been vaccinated, 51 had the disease in its confluent form, in 183 it was discrete, and in 7 modified; while of the 58 vaccinated, it was confluent only in 5, discrete in 34, and modified in 19. Of the vaccinated with cicatrices, 2 had the confluent form, 14 the discrete, and 18 the modified. Of those with imperfect cicatrices, none confluent, 11 discrete, and one modified; in those without cicatrices, 3 confluent, 9 discrete, and none modified.

*Proportion of Confluent, Discrete, and Modified Cases.*

| | Cases. | | Confluent. | | Discrete. | | Modified. |
|---|---|---|---|---|---|---|---|
| Unvaccinated - - - | 241 | .. | 51 | .. | 183 | .. | 7 |
| Vaccinated - - - | 58 | .. | 5 | .. | 34 | .. | 19 |
| Vaccinated, with good cicatrix - | 34 | .. | 2 | .. | 14 | .. | 18 |
| ———— imperfect cicatrix - | 12 | .. | 0 | .. | 11 | .. | 1 |
| ———— without cicatrix - | 12 | .. | 3 | .. | 9 | .. | 0 |

With regard to the effect of race on the mortality, it appears, that of the unvaccinated there were 5 whites, with one death; 98 of the coloured race, with 18 deaths; and 138 negroes, with 26 deaths. The ratio, therefore, in all races, appears to have been the same. It is true that both the deaths which took place among the 58 vaccinated, occurred in the black race, but the circumstances attending these have already been explained. Of the vaccinated, 16 were children under 14 years of age, all of whom, with two exceptions, had cicatrices more or less perfect; of the adults some had been vaccinated within a few years, but the majority for a longer period; but Dr. Bowerbank did not observe that the cases of small-pox which occurred in persons recently vaccinated were milder than in those in whom the vaccination was of older date.

Dr. Turner of Spanish Town has stated the result of 307 cases of small-pox attended by him : 236 of these had neither been vaccinated nor inoculated, and 30 of them died; 62 cases, with 2 deaths, occurred in persons who had been vaccinated; 3 adults, who had had small-pox naturally in infancy, contracted it again, and of them one died; of 6 adults who had had small-pox by inoculation in childhood, and were again attacked, none died. Of the unvaccinated 21 were infants, of whom 13 died; 58 were under 12 years of age, of whom 3 died; and 157 were adults, of whom 14 died. Of the vaccinated, 5 were infants,* of whom one died; 24 were under 12, of whom none died; and 33 were adults, of whom one died. It will be seen by Dr. Turner's statement that nine of the persons attended by him had had small-pox previously, either naturally or by inoculation; and I have already observed, that Dr. Bowerbank saw two cases in persons who had been inoculated. Dr. Clarke also saw cases in persons who had the cicatrices of inoculation. Dr. Chamberlaine of Kingston, both in the epidemic of 1831 and recently, saw various cases occurring in persons who had had the disease previously, either naturally or by inoculation. Dr. Dalrymple of Trelawney saw no case of secondary small-

* These were probably cases in which vaccination had not been performed until after the variolous poison had been received into the system. E. C. S.

pox, but several in persons who had been vaccinated,—all, however, mild, and without a fatal result. Dr. McGrath of Kingston has had, in his own person, the confluent form of small-pox succeeding to inoculation.

With all this evidence, we cannot be surprised to find the medical practitioners of Jamaica expressing, without exception, their unabated confidence in the protective value of vaccination, and, with a single exception, their opinion that inoculation is inexpedient and unjustifiable. Even the exception is more apparent than real; because *he* would limit the practice to those cases in which a large number of persons susceptible of small-pox are collected together, *and no supply of reliable vaccine lymph can be procured.*

It had been my wish to compare the observations I have now detailed with those made by Dr. Kinnis in Ceylon, Mr. Gardner in the Mauritius, and with results observed in India and in other places, with the view of determining, on the widest attainable basis, the protective value of vaccination in hot climates, and of testing the truth or falsehood of the extraordinary statement made three or four years ago, in a work generally considered to be of authority, and intended to inform the minds and direct the practice of the rising generation of medical men, that "in hot climates and dark varieties of the human species, vaccination had been *demonstrated* to be inefficacious," whilst "inoculation had been found, and still was found, the most certain protection from the severer distemper, and from epidemic outbreaks." But some work which could not be delayed, recently undertaken for the Society, has occupied so much of my time, that I have been unable to fulfil this part of my task. I cannot but think, however, that the history of this epidemic forms in itself a useful contribution to our knowledge on this subject, and, so far as the history of one epidemic can do, tends entirely to negative the bold assertions I have just read; and while it will be seen that the practitioners of Jamaica have arrived at a conclusion diametrically opposite to that so strongly stated by Dr. Copland, I think it will be conceded that they have proved that they are not liable to the imputation which he has sweepingly thrown on all who venture to dispute his dogma, of being "overborne by the unworthy influences of authority and prejudice," and that they may even claim a place in that higher class of whom he says, that "they observe and think for themselves."

